# Genome-Guided Discovery of Pretilactam from *Actinosynnema pretiosum* ATCC 31565

**DOI:** 10.3390/molecules24122281

**Published:** 2019-06-19

**Authors:** Jing Wang, Xiaowen Hu, Guizhi Sun, Linli Li, Bingya Jiang, Shufen Li, Liping Bai, Hongyu Liu, Liyan Yu, Linzhuan Wu

**Affiliations:** 1 NHC Key Laboratory of Biotechnology of Antibiotics, Institute of Medicinal Biotechnology, Chinese Academy of Medical Sciences and Peking Union Medical College, Beijing 100050, China; wjing91@163.com (J.W.); dreadless@126.com (X.H.); 201421150020@mail.bnu.edu.cn (G.S.); lily1914@126.com (L.L.); lisf0229@163.com (S.L.); lipingbai1973@163.com (L.B.); cpcc126@126.com (H.L.); yly@cpcc.ac.cn (L.Y.); 2 CAMS Key Laboratory of Synthetic Biology for Drug Innovation, Institute of Medicinal Biotechnology, Chinese Academy of Medical Sciences and Peking Union Medical College, Beijing 100050, China

**Keywords:** *Actinosynnema pretiosum* ATCC 31565, polyene macrolactam, pretilactam, antiSMASH

## Abstract

*Actinosynnema* is a small but well-known genus of actinomycetes for production of ansamitocin, the payload component of antibody-drug conjugates against cancers. However, the secondary metabolite production profile of *Actinosynnema pretiosum* ATCC 31565, the most famous producer of ansamitocin, has never been fully explored. Our antiSMASH analysis of the genomic DNA of *Actinosynnema pretiosum* ATCC 31565 revealed a NRPS–PKS gene cluster for polyene macrolactam. The gene cluster is very similar to gene clusters for mirilactam and salinilactam, two 26-membered polyene macrolactams from *Actinosynnema mirum* and *Salinispora tropica*, respectively. Guided by this bioinformatics prediction, we characterized a novel 26-membered polyene macrolactam from *Actinosynnema pretiosum* ATCC 31565 and designated it pretilactam. The structure of pretilactam was elucidated by a comprehensive analysis of HRMS, 1D and 2D-NMR, with absolute configuration of chiral carbons predicted bioinformatically. Pretilactam features a dihydroxy tetrahydropyran moiety, and has a hexaene unit and a diene unit as its polyene system. A preliminary antibacterial assay indicated that pretilactam is inactive against *Bacillus subtilis* and *Candida albicans*.

## 1. Introduction

Genome-guided discovery of secondary metabolites is a powerful strategy for rational identification of novel compounds from microorganisms [1,2]. Recent advances in DNA sequencing technologies allow rapid and low-cost sequencing of microbial genomic DNA, and bioinformatics tools (antiSMASH, ClusterMine360, and NORINE, etc.) are also developed for detection of various gene clusters for secondary metabolites (even their structures) in microbial genomes, which greatly facilitate the de-replication of known metabolites and targeted mining of new ones in the chemistry study of secondary metabolites produced by microorganisms [3,4,5]. The gene clusters predicted by the comprehensive antiSMASH may provide valuable information about secondary metabolite production profiles of microbial strains analyzed.

Polyene macrolactams are a growing family of microbial secondary metabolites biosynthesized by NRPS–PKS (nonribosomal peptide synthase–polyketide synthase) complexes. Quite a few polyene macrolactams, together with their (putative) gene clusters, have been identified from actinomycetes in the past decade, such as auroramycin from *Streptomyces*, micromonolactam and lobosamide from *Micromonospora*, mirilactam from *Actinosynnema mirum*, and salinilactam from *Salinispora tropica* [6,7,8,9,10,11,12,13,14]. It is worthy to note that some of these polyene macrolactams display antibacterial, antiprotozoal, or cytotoxic activities [9,10,11].

*Actinosynnema* is a small but well-known genus of actinomycetes for its production of ansamitocin, the payload component of antibody-drug conjugates against cancers. *Actinosynnema pretiosum* ATCC 31565 (hereafter in brief ATCC 31565) is the most famous ansamitocin producer [14,15,16,17]. However, the secondary metabolite production profile of ATCC 31565 has never been fully explored, although it was reported to produce secondary metabolites dnacin (a naphthyridinomycin-type antitumor antibiotic) and actinosynneptide (a cytotoxic hexapeptide) [18,19,20].

To get a deep insight into the secondary metabolite biosynthetic potential of ATCC 31565, we sequenced its genomic DNA to direct our chemical investigation of secondary metabolites for new compound(s). Herein, the genome-guided identification of a novel 26-membered polyene macrolactam from ATCC 31565 is described as below.

## 2. Results and Discussion

### 2.1. ATCC 31565 Contains a Gene Cluster for Polyene Macrolactam

**Figure 1:**
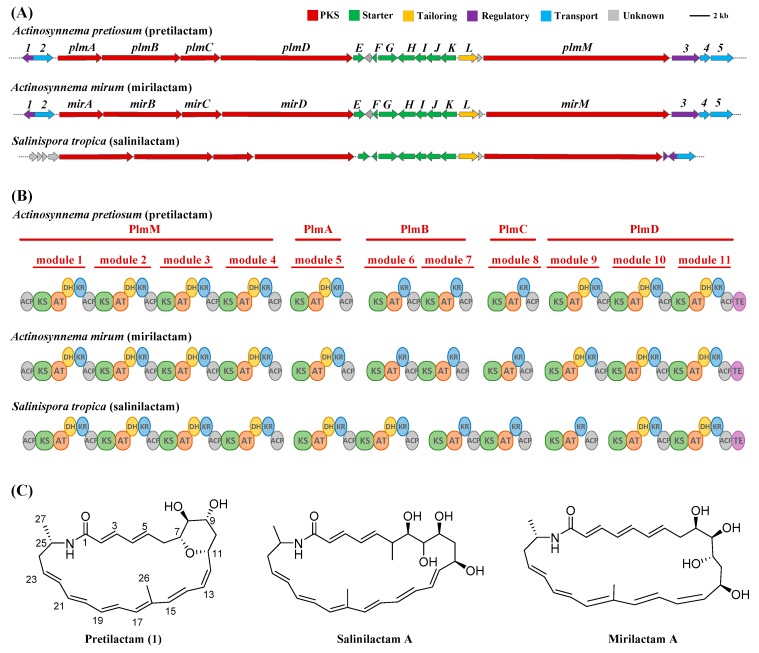
(**A**) The *plm* gene cluster for a putative polyene macrolactam (pretilactam, **1**) from *Actinosynnema pretiosum* ATCC 31565 and its comparison with gene clusters for mirilactam from *Actinosynnema mirum* and salinilactam from *Salinispora tropica*. (**B**) Alignment of PKS domains for biosynthesis of the putative polyene macrolactam (pretilactam, **1**), mirilactam and salinilactam. ACP, acyl carrier protein; AT, acyltransferase; DH, dehydrotase; KR, ketoreductase; KS, ketosynthase. (**C**) Structures of pretilactam (**1**), salinilactam A, and mirilactam A.

Genome sequencing and assembly resulted in a linear chromosome DNA of 8,131,271 bp for ATCC 31565. AntiSMASH analysis of the chromosome DNA revealed over 20 gene clusters for various secondary metabolites, including the expected gene cluster for ansamitocin biosynthesis. Among them, an 80 kb gene cluster for NRPS–PKS showed very high percentages of similar genes to mirilactam and salinilactam gene clusters. Specifically, the gene cluster contained 5 genes for type I modular polyketide synthases (PKS), 1 gene for cytochrome P450 oxidase, and a set of 7 genes for 3-aminobutyrate (the starter of some polyene macrolactams) biosynthesis. The enzymes/proteins encoded by these genes showed ≥96% amino acid sequence identities to those for mirilactam biosynthesis. In particular, the 11 modules of the five PKSs revealed the same or very similar domain organization to PKSs for mirilactam or salinilactam biosynthesis (Figure 1A,B, Table 1). Thus, the gene cluster should be responsible for the biosynthesis of polyene macrolactam(s) very similar or identical to mirilactam or salinilactam. The gene cluster was designated as *plm*, and deposited in GenBank with the accession number MK341065.

### 2.2. The Putative Polyene Macrolactam(s) was Detected by LC-MS

**Figure 2:**
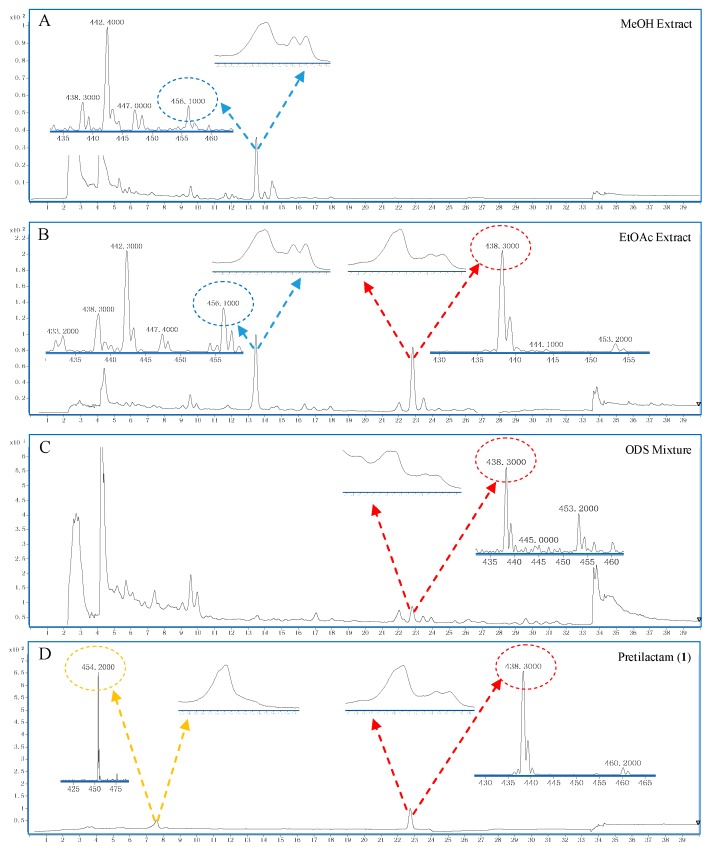
LC-MS for putative polyene macrolactam and the purified polyene macrolactam pretilactam (**1**). (**A**) A molecular ion *m*/*z* 456 [M + H]^+^ appeared at 13.5 min in the MeOH extract of ATCC 31565. (**B**) Two molecular ions *m*/*z* 456 [M + H]^+^ and 438 [M + H]^+^ appeared at 13.5 and 22.7 min, respectively, in the EtOAc extract of ATCC 31565. (**C**) Only the molecular ion *m*/*z* 438 [M + H]^+^ appeared at 22.7 min in the ODS mixture before column fractionation. (**D**) A molecular ion *m*/*z* 438 [M + H]^+^ appeared at 22.7 min for the purified pretilactam (**1**). The molecular ion *m*/*z* 454 [M + H]^+^ at 7.6 min should be a degradation molecule due to incubation of the purified pretilactam (**1**) at 4 °C for 24 h before analysis.

The fermentation culture of ATCC 31565 was extracted with methanol or ethyl acetate, and the extracts were analyzed by LC-MS. The LC traces revealed peaks with UV absorption profiles very similar to mirilactam A (Figure 2). The hyphenated MS spectra of these peaks displayed molecular ions *m*/*z* 456 (the same as that of mirilactam A) and/or 438 [M + H]^+^. Thus, putative polyene macrolactams were found in ATCC 31565.

### 2.3. Structure of the Polyene Macrolactam (**1**, Pretilactam) was Elucidated by NMR

Compound **1** is a white amorphous solid. Its molecular formula was established as C_27_H_35_O_4_N by HRMS (Figure S1), indicating 11 degrees of unsaturation. The ^13^C-NMR, DEPT, and HSQC spectra (Figures S2–S8) of **1** differentiated 27 carbon atoms, including 1 carbonyl group (δ_C_ 166.2), 1 nonprotonated olefinic carbon (δ_C_ 135.7), 20 methine groups (including 15 olefinic groups δ_C_ 124.7–139.7, 4 oxymethine groups δ_C_ 66.8–71.4, and 1 nitrogen-substituted methine group δ_C_ 44.7), 3 methylene groups δ_C_ 34.8–42.2, and 2 methyl groups δ_C_ 13.1–21.3. The presence of many olefinic groups indicated that **1** is a polyene.

The COSY and TOCSY spectra of **1** revealed two spin systems. The first spin system (C-2 to C-15) contained 4 oxymethines, 2 methylenes, and two diene fragments (C-2 to C-5, C-12 to C-15). The COSY correlations between H-8 (δ_H_ 3.13) and an active hydrogen (δ_H_ 4.58, 8-OH), and between H-9 (δ_H_ 3.90) and another active hydrogen (δ_H_ 4.53, 10-OH), established two hydroxymethines: C-8 (δ_C_ 71.4) and C-9 (δ_C_ 67.0). The chemical shifts of δ_C_ 71.0 (δ_H_ 3.62) and δ_C_ 66.8 (δ_H_ 4.58) disclosed the other two oxymethines at C-7 and C-11, respectively. The HMBC correlation from H-7 to C-11 indicated that the two oxymethines must share an oxygen (as a bridge to join C-7 and C-11), and this was also supported by the molecular formula requirement of **1**, i.e., two oxygens in hydroxys, one oxygen in carbonyl, thus only one oxygen left for two oxymethines. Therefore, a dihydroxy tetrahydropyran moiety was determined in **1**. The HMBC correlations from H-2 and H-3 to the carbonyl at δ_C_ 166.2 and NOESY correlation between H-2 and NH proved that the diene fragment of C-2 to C-5 conjugated with an amide group. The HMBC correlations from H-14 (δ_H_ 6.70) to the nonprotonated olefinic carbon δ_C_ 135.7 (C-16), H_3_-26 (δ_H_ 1.70) to the olefinic carbon C-17 (δ_C_ 130.9), and NOESY correlation between H-14 and C-26 methyl group (δ_C_ 13.1, δ_H_ 1.70 s) further extended the diene fragment of C-12 to C15 to a triene fragment of C-12 to C-17.

The second spin system (C-27, C-25 to C-22) contained a methyl, a methine, a methylene, and two sp2 methines. The COSY correlation between the methine (δ_H_ 3.82, H-25) and an active hydrogen (δ_H_ 7.35, -NH-), together with the chemical shifts of the methine (δ_H_ 3.82, δ_C_ 44.7), demonstrated that C-25 connected to the nitrogen atom. The HMBC correlation from NH group (δ_C_ 7.35) to C-1 (δ_C_ 166.2), as well as only one nitrogen atom in the molecular formula of **1**, determined the connection of the carbonyl C-1 with C-25 via NH.

**Figure 3:**
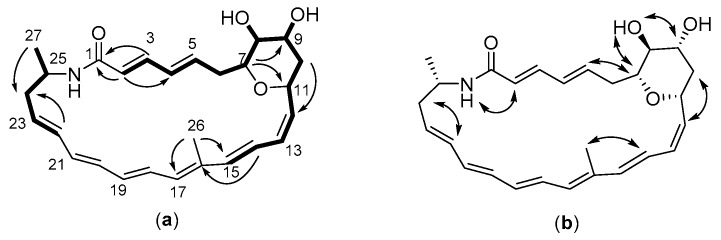
(**a**) ^1^H-^1^H COSY and key HMBC correlations of pretilactam (**1**). (**b**) Key NOESY correlations of pretilactam (**1**).

Four sp2-hybridized methine carbons (δ_C_ 128.7, 131.0, 132.1, and 139.0) could not be assigned due to the imperfectness of 1D- and 2D-NMR signals, with their corresponding proton signals overlapped except the δ_H_ 6.19 for δ_C_ 128.7. However, based on the unsaturation degrees of **1** and UV absorption profile (maximal absorption at 301, 343, and 364 nm suggested a hexaene unit; Figure 2), the four sp2-hybridized methines must form a diene fragment that connected the above two spin systems at C-17 and C-22 to form a hexaene unit, thereby completing the 26-membered polyene macrolactam of **1** (Figure 3).

The geometries of five C-C double bonds in **1** were assigned to be 2E, 12Z, 14Z, 16E, and 22E based on their vicinal coupling constants (*J*_H2-H3_ = 14.4 Hz, *J*_H12-H13_ = 11.2 Hz, and *J*_H14-H15_ = 15.2 Hz, *J*_H18-H19_ = 14.4 Hz, *J*_H22-H23_ = 14.4 Hz) and the NOESY correlations of H-14/H-26. Owing to the overlaps of proton signals and NOESY correlations, the geometries of C-C double bonds at 4(5), 18(19), and 20(21) in **1** could not be determined. Thus, the planar structure of **1** was determined by NMR interpretation. Compound **1** was designated as pretilactam. Its NMR data were assigned in Table 2.

The absolute configuration of chiral carbons in pretilactam may be predicted bioinformatically. The chiral C-9 and C-11 should take the same *S* configuration because substrate predictions for KR stereochemistry in module 8 and module 7 (Figure 1B) were both A1 by antiSMASH [3,21]. Chiral C-25 should take the same *S* configuration as in mirilactam A because the seven genes for 3-aminobutyrate biosynthesis from ATCC 31565 could be regarded as the same with those from *Actinosynnema mirum* (based on ≥96% amino acid sequence identities for each enzyme or protein encoded by the 7 genes) [6]. Besides, the *S* configuration of C-25 was also supported by the stereochemistry of mirilactams C-E [12]. The H-7, OH-8, and H-9 were strongly suggested to locate on the same surface of the tetrahydropyran ring by NOESY correlations of H-7/OH-8 and OH-8/H-9. Thus, chiral C-7 and C-8 should take the same *R* configuration (Figure 1C).

The structure of pretilactam (**1**) elucidated by NMR matches rather well with the structure predicted by its gene cluster, except the following three minor points. First, the formation of C-C double bond 12(13) requires an active DH domain in module 6. Second, the formation of C-6(hydroxy),7 single bond demands an inactive DH domain in module 9. Third, the substrate predictions of acyltransferase domains AT2, AT3, AT4, and AT8 are not accurate. Similar disagreements have occurred many times in structure prediction by other type I modular PKSs [6,22,23,24,25].

### 2.4. The Dihydroxy Tetrahydropyran Ring Might Come from Dehydration of a Tetrahydroxy Pentane Unit in Pretilactam Biosynthesis

**Figure 4:**
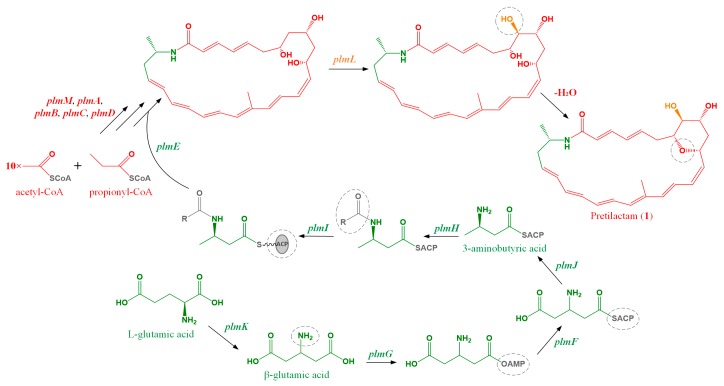
The plausible biosynthetic pathway of pretilactam (**1**).

Pretilactam features a dihydroxy tetrahydropyran ring, which might be formed by spontaneous dehydration of a tetrahydroxy pentane unit in the final stage of pretilactam biosynthesis. Based on present understanding of microbial polyene macrolactams biosynthesis, we proposed a plausible biosynthetic pathway for pretilactam (Figure 4).

### 2.5. Pretilactam was Inactive Against Bacillus Subtilis and Candida Albicans

A preliminary biological activity assay indicated that pretilactam showed no growth inhibition against *Bacillus subtilis* CMCC 63501 and *Candida albicans* ATCC 10231 at an amount of ca. 25 μg/paper disk. Due to unstable property of pretilactam (and insufficient amount of pretilactam), other biological activity assays of pretilatam were not conducted.

## 3. Discussion

Pretilactam is a novel 26-membered polyene macrolactam. Its discovery indicates that ATCC 31565, the famous ansamitocin producer, is a talent strain for various secondary metabolites. 

It is rather challenging to determine the absolute configuration of many polyene macrolactams because of their unstable property and unassigned E/Z geometries of some C-C double bonds. We found that pretilactam was sensitive to light, air, and acidic pH, and changed slowly in organic solvents such as methanol, acetonitrile, and DMSO. In addition, pretilactam has three C-C double bonds unassigned of their E/Z geometries. It is, therefore, very difficult to determine the absolute configuration of chiral carbons in pretilactam by chemical analysis.

The *plm* is believed to be responsible for pretilactam biosynthesis because it is the only candidate gene cluster for polyene macrolactam biosynthesis by antiSMASH of the genomic DNA of ATCC 31565. Still, genetic manipulation of *plm* should be carried out to confirm its biological function experimentally. Besides, the biological activity of pretilactam needs to be further explored in the future.

## 4. Materials and Methods

### 4.1. Cultivation of ATCC 31565

Frozen stock spores of ATCC 31565 were inoculated onto ISP2 (glucose 4.0 g/L, malt extract 10.0 g/L, yeast extract 4.0 g/L, agar 15.0 g/L) plates and incubated at 28 °C for 5–7 days to develop mycelia with fresh spores. The fresh spores were collected and spread onto fermentation plates (dextrin 50.0 g/L, maltose 20.0 g/L, Pharmamedia 25.0 g/L, corn steep liquor 1.5 g/L, K_2_HPO_4_, 0.5 g/L, NaCl 2.0 g/L, agar 20.0 g/L, pH 7.0), then incubated at 28 °C for 8–10 days to produce mycelia lawns.

### 4.2. Genomic DNA Sequence Analysis

The genomic DNA of ATCC 31565 was extracted from mycelia using DNeasy blood and tissue kit from QIAGEN (Hilden, Germany). The DNA was sequenced on Illumina NGS and PacBio RSII with P4/C6 chemistry using the SMRTbell template prep kit v1.0 (Pacific Biosciences, CA, USA). Sequence data were processed and assembled using HGAP3, and sequence assembly was polished with the Quiver algorithm implemented in the PacBio SMRT analysis suite v2.3.0 (Pacific Biosciences, CA, USA).

### 4.3. LC-MS for Polyene Macrolactam

Fermentation culture of ATCC 31565 was extracted with an equal volume of MeOH or EtOAc. The MeOH or EtOAc extract (50 mL) was vacuum-dried then re-dissolved in 1.0 mL MeOH. An amount of 10 μL MeOH solution was used for LC-MS.

The above EtOAc extract of ATCC 31565 was mixed with ODS (12 nm, S-50 μm, AAG12S50, YMC) at a proportion of 1:1.5, then vacuum-dried at room temperature. The dried mixture was re-extracted with MeOH, and the MeOH solution was used for LC-MS.

A purified sample of pretilactam (**1**), dissolved in MeCN and stored at 4 °C for 24 h, was used for LC-MS.

LC-MS parameters: For LC analysis, an Agilent 1100 was used, with a Diamonsil C18 (4.6 × 250 mm, 5 μm); mobile phase CH_3_CN-H_2_O, 1.0 mL/min, 30–50% in 30 min; wavelength 300 nm; 25 °C. For MS analysis, an Agilent 6410 QQQ was used, with MS2 scan type; fragmentor 60 V; positive polarity.

### 4.4. Isolation of Polyene Macrolactam (Pretilactam, ***1***)

A large-scale cultivation of ATCC 31565 was performed to obtain enough material for isolation and characterization of the putative polyene macrolactam(s). The fermentation cultures (50 L; from ca. 1000 plates, 50 mL/plate) were pooled and extracted with EtOAc. The organic layer was collected and evaporated under reduced pressure, which gave 175 g of oily brown residue. The residue was loaded on an ODS column (12 nm, S-50 μm, AAG12S50, YMC; 460 × 49 mm), and fractionated by a step-wise gradient (20%, 30%, 40%, 50%, and 100%) of ethanol-H_2_O. Fraction 4 from 50% ethanol-H_2_O elution was evaporated under reduced pressure, which afforded 257.2 mg crude preparation for the putative polyene macrolactam(s). The crude preparation was subjected to preparative HPLC (YMC-Pack ODS-A, 250 × 20 mm, S-5 μm, 12 nm; 50–80% CH_3_CN-H_2_O, 25 min), which gave 11.2 mg refined preparation of **1** after evaporation. The refined preparation was subjected to semi-preparative HPLC (YMC-Pack ODS-A, 250 × 10 mm, S-5 μm, 12 nm; 78% CH_3_OH-H_2_O) for a final polishing, which yielded 4.1 mg pure preparation of **1** (with purity at 98% by HPLC analysis). The isolation process was conducted under dark, and dried samples in the purification process were stored in glass vials filled with N_2_ gas, as polyene macrolactams are sensitive to light and O_2_.

### 4.5. NMR Assay

^1^H and ^13^C-NMR spectra data were obtained at 800 and 200 MHz, respectively, on a BRUKER AVANCE III 800 spectrometer, and measured in DMSO-*d*_6_ at room temperature.

### 4.6. Assay of Pretilactam (***1***) Against Bacillus Subtilis CMCC 63501 and Candida Albicans ATCC 10231

A stock suspension (5–10 μL) of *Candida albicans* ATCC 10231 (or *Bacillus cereus* CMCC 63501) was inoculated into LB broth (100 mL in a 500 mL flask) for shaking at 37 °C overnight. The fresh culture of *Candida albicans* ATCC 10,231 (or *Bacillus cereus* CMCC 63501) was added into LB broth (with agar at 1.5%, 45 °C) at a proportion of 1.0% and mixed well, then poured into plates (12.0 mL for each plate, with a diameter of 8.5 cm; 10^6−7^ CFU/mL). After solidification, the LB agar plates were ready for bioassay uses.

Filter paper disks (diameter 6.0 mm) loaded with ca. 6.25, 12.5, and 25 μg pretilactam (**1**) per paper disk were overlaid on the LB agar plates, then incubated at 37 °C for 12~15 h for microbial growth and inhibition zone formation.

Pretilactam (**1**): amorphous white powder; UV (MeOH) *λ*_max_ (log *ε*) = 298 (4.54), 345 (3.22), 362 (3.09). IR (MeOH, cm^−1^): 3344.9, 2919.8, 2854.2, 1730.2, 1643.9, 1608.8, 1430.6, 1373.3, 1318.1, 1258.7, 1163.0, 1110.3, 1059.4, 898.0, 801.2, 662.9, 615.3. ^1^H-NMR and ^13^C-NMR data, Table 2; HRESIMS *m*/*z* 438.2646 [M + H]^+^ (calculated for C_27_H_36_O_4_N, 438.2639).

## Figures and Tables

**Table 1:** Predicted function of ORFs from *plm* gene cluster for a putative polyene macrolactam (pretilactam, **1**) in ATCC 31565.

ORF	Size (aa)	Homologue [Accession No./Strain]	Identity/Positive	Predicted Function
*plm1*	210	TetR/AcrR family [ACU36614.1/*Actinosynnema mirum* DSM 43827]	92/96	transcriptional regulator
*plm2*	613	major facilitator superfamily MFS_1 [ACU36615.1/*Actinosynnema mirum* DSM 43827]	95/96	MFS transporter
*plmA*	1816	short-chain dehydrogenase/reductase SDR [ACU36616.1/*Actinosynnema mirum* DSM 43827]	97/98	type I modular PKS
*plmB*	3092	short-chain dehydrogenase/reductase SDR [ACU36617.1/*Actinosynnema mirum* DSM 43827]	96/97	type I modular PKS
*plmC*	1606	short-chain dehydrogenase/reductase SDR [ACU36618.1/*Actinosynnema mirum* DSM 43827]	96/97	type I modular PKS
*plmD*	5400	short-chain dehydrogenase/reductase SDR [ACU36619.1/*Actinosynnema mirum* DSM 43827]	96/97	type I modular PKS
*plmE*	296	proline-specific peptidase [ACU36620.1/*Actinosynnema mirum* DSM 43827]	96/97	peptidase
*plmF*	82	phosphopantetheine-binding protein [ACU36622.1/*Actinosynnema mirum* DSM 43827]	100/100	phosphopantetheine-binding protein
*plmG*	529	AMP-dependent synthetase and ligase [ACU36623.1/*Actinosynnema mirum* DSM 43827]	96/97	synthetase and ligase
*plmH*	507	AMP-dependent synthetase and ligase [ACU36624.1/*Actinosynnema mirum* DSM 43827]	98/98	synthetase and ligase
*plmI*	308	S-malonyltransferase-like protein [ ACU36625.1/*Actinosynnema mirum* DSM 43827]	98/98	ACP S-malonyl transferase
*plmJ*	419	pyridoxal phosphate-dependent aminotransferase [ACU36626.1/*Actinosynnema mirum* DSM 43827]	97/98	aminotransferase
*plmK*	450	l-lysine 2,3-aminomutase [ACU36627.1/*Actinosynnema mirum* DSM 43827]	99/99	glutamate 2,3-aminomutase
*plmL*	395	cytochrome P450 [ACU36628.1/*Actinosynnema mirum* DSM 43827]	98/99	cytochrome P450 oxidase
*plmM*	7110	short-chain dehydrogenase/reductase SDR [ACU36630.1/*Actinosynnema mirum* DSM 43827]	97/98	type I modular PKS
*plm3*	931	transcriptional regulator, LuxR family [ACU36631.1/*Actinosynnema mirum* DSM 43827]	98/98	transcriptional regulator
*plm4*	279	ABC transporter related [ACU36632.1/*Actinosynnema mirum* DSM 43827]	99/100	ATP-binding protein
*plm5*	855	FtsX-like permease family protein [ACU36633.1/*Actinosynnema mirum* DSM 43827]	98/99	FtsX-like permease family

**Table 2:** NMR data of pretilactam (**1**).

Position	δ_H_ (mult., *J* in Hz)	δ_C_	^1^H-^1^H COSY	HMBC	NOESY
1		166.2			
2	5.84 (d, 14.4)	124.7	H-3	C-1, C-4	NH
3	6.52 (dd, 14.4, 10.4)	139.7	H-2, H-4	C-1	
4	6.11 (ovl ^a^)	127.8	H-5		
5	5.54 (m)	138.3	H-4, H-6		H-7
6	2.55 (m), 2.10 (m)	34.8	H-5, H-7	C-5, C-7	
7	3.62 (t, 8.8)	71.0	H-6, H-8	C-6, C-8, C-9, C-11	8-OH
8	3.13 (br.t, 6.4)	71.4	H-7, H-9, OH-8		
9	3.90 (brs)	67.0	H-8, H-10, OH-9		8-OH
10	1.81 (brs)	35.5	H-9, H-11	C-11, C-12	H-12
11	4.58 (ovl ^a^)	66.8	H-10, H-12		
12	5.85 (m)	133.1	H-11, H-13	C-11	
13	6.16 (t, 11.2)	132.9	H-12, H-14		
14	6.70 (dd, 14.4, 12.0)	126.0	H-13, H-15	C-16	H_3_-26
15	6.24 (d, 15.2)	136.6	H-14	C-26	
16		135.7			
17	6.10 (ovl ^a^)	130.9 ^a^			
18	6.19 (dd, 14.4, 12.8)	128.7			
19	6.10 (ovl ^a^)	131.0 ^a^			
20	6.10 (ovl ^a^)	132.1 ^a^			
21	6.10 (ovl ^a^)	139.0			
22	5.90 (dd, 14.4, 11.2)	134.8	H-23	C-24	H-24
23	5.46 (m)	130.7	H-22, H-24		
24	2.33 (m), 1.69 (ovl ^a^)	42.2	H-23, H-25	C-25, C-23	
25	3.82 (brs)	44.7	H-24, H-27, NH		
26	1.70 (s)	13.1		C-16, C-17	
27	1.12 (d, 6.4)	21.3	H-25	C-24, C-25	
NH	7.35 (d, 9.6)		H-25	C-1, C-25	H-2
8-OH	4.58 (d, 6.4)		H-8		H-7, H-9
9-OH	4.53 (d, 2.4)		H-9		

^a^ Overlapped with other peaks.

## Supplementary Materials

The supplementary materials are available online.
