# Supplementary material for: Genome-Guided Discovery of Pretilactam from Actinosynnema pretiosum ATCC 31565
Source: Molecules. 2019 Jun 19;24(12):2281. doi: 10.3390/molecules24122281 (PMC6631532; doi:10.3390/molecules24122281)
Supplement: Supplementary file 1 [file molecules-24-02281-s001.pdf]

Supplementary material

Genome-Guided discovery of pretilactam from *Actinosynnema pretiosum* ATCC 31565

Jing Wang, Xiaowen Hu, Guizhi Sun, Linli Li, Bingya Jiang, Shufen Li, Liping Bai, Hongyu Liu, Liyan Yu, Linzhuan Wu**

NHC Key Laboratory of Biotechnology of Antibiotics, CAMS Key Laboratory of Synthetic Biology for Drug Innovation, Institute of Medicinal Biotechnology, Chinese Academy of Medical Sciences and Peking Union Medical College, Beijing 100050, People's Republic of China

*To whom correspondence should be addressed. Tel: +86-10-63165283. Fax: +86-10-63017302. E-mail: jiangbingya@163.com or wulinzhuan@imb.pumc.edu.cn.

Contents

Figure S1 AntiSMASH of the genomic DNA of ATCC 31565……………………………… ……S3

Figure S2 HR(+)ESIMS of pretilactam (**1**)……………………………………………………S4

Figure S3 ^{1}H NMR spectrum (800 MHz) of pretilactam (**1**) in DMSO-d_6……………………S4

Figure S4 ^{13}C NMR spectrum (200 MHz) of pretilactam (**1**) in DMSO-d_6…………………...S5

Figure S5 ^{1}H-^{1}H COSY spectrum (800 MHz) of pretilactam (**1**) in DMSO-d_6……………..…S5

Figure S6 HSQC spectrum (200 MHz) of pretilactam (**1**) in DMSO-d_6………………………S6

Figure S7 HMBC spectrum (200 MHz) of pretilactam (**1**) in DMSO-d_6………………...……S6

Figure S8 TOCSY spectrum (800 MHz) of pretilactam (**1**) in DMSO-d_6……………………..S7

Figure S9 NOESY spectrum (800 MHz) of pretilactam (**1**) in DMSO-d_6……………………..S7

Figure S10 UV spectrum of pretilactam (**1**)……………………………………...………....S8

Figure S11 IR spectrum of pretilactam (**1**)……………………………………...….……..S8

Identified secondary metabolite regions

Region	Type	From	To	Most similar known cluster		Similarity	MIBiG BGC-ID
The following regions are from record NODE_1:							
Region 1	Terpene	46280	66796	SF2575	polyketide	6%	BGC0000269
Region 2	Lanthipeptide	230028	253096	Tetronasin	polyketide	9%	BGC0000163
Region 3	Terpene	3209146	3229317	Geosmin	other	100%	BGC0001181
Region 4	Bacteriocin	3837575	3848375				
Region 5	Indole	4468806	4489927	Frankiamicin	polyketide	14%	BGC0001197
Region 6	Nrpsfragment-Otherks-T1pks	4936116	4980643				
Region 7	Nrps-T1pks	5169664	5327702	Salinilactam	polyketide	88%	BGC0000142
Region 8	Nucleoside	5384654	5405622	Tunicamycin	other	64%	BGC0000880
Region 9	Nrps-T1pks	5652921	5732628	Tirandamycin	hybrid	40%	BGC0001052
Region 10	Otherks	5814814	5854503	Calcimycin	polyketide	13%	BGC0000032
Region 11	Nrpsfragment-T1pks	5878730	5975542	Ansamitocin	polyketide	97%	BGC0000020
Region 12	Lassopeptide	6034265	6056728	SF2575	polyketide	4%	BGC0000269
Region 13	Nrps-Phosphonate-T1pks	6066797	6154441	Himastatin	NRPS	8%	BGC0001117
Region 14	Nrps-Otherks	6328654	6476784	Naphthyridinomycin	NRPS	60%	BGC0000394
Region 15	Terpene	6599385	6618894	Isorenieratene	terpene	85%	BGC0000664
Region 16	Thiopeptide	6643965	6673418				
Region 17	T1pks	6679293	6719988	Coelimycin	polyketide	8%	BGC0000038
Region 18	T1pks	6828788	6884394				
Region 19	T1pks	6917680	6962500	Kedarcidin	polyketide	6%	BGC0000081
Region 20	T1pks	7248874	7310026	Spinosad	polyketide	8%	BGC0000148
Region 21	Terpene	7319928	7341610	Brasilicardin A	terpene	45%	BGC0000632
Region 22	Nrps	7369240	7462182	Marformycins	NRPS	12%	BGC0001214
Region 23	Nrps	7499669	7542655				
Region 24	Nrpsfragment-Other-T1pks	7542698	7611214	Lasalocid	polyketide	16%	BGC0000087
Region 25	T1pks	7645186	7703975	Coelimycin	polyketide	25%	BGC0000038
Region 26	Oligosaccharide	7915377	7958960				
Region 27	Nrps	8017917	8071251	Erythrochelin	NRPS	85%	BGC0000349

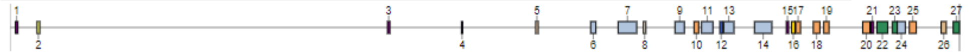

Figure S1 AntiSMASH of the genomic DNA of ATCC 31565

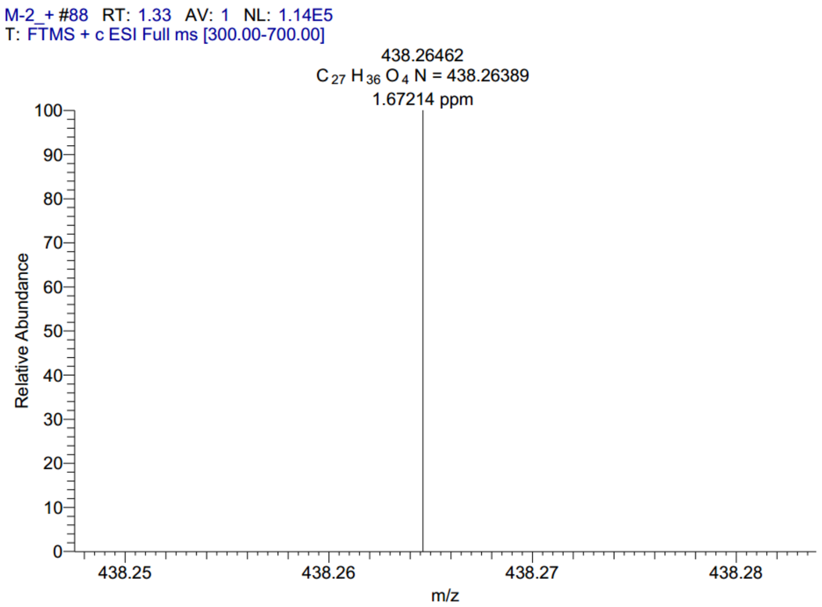

Figure S2 HR(+)ESIMS of pretilactam (**1**)

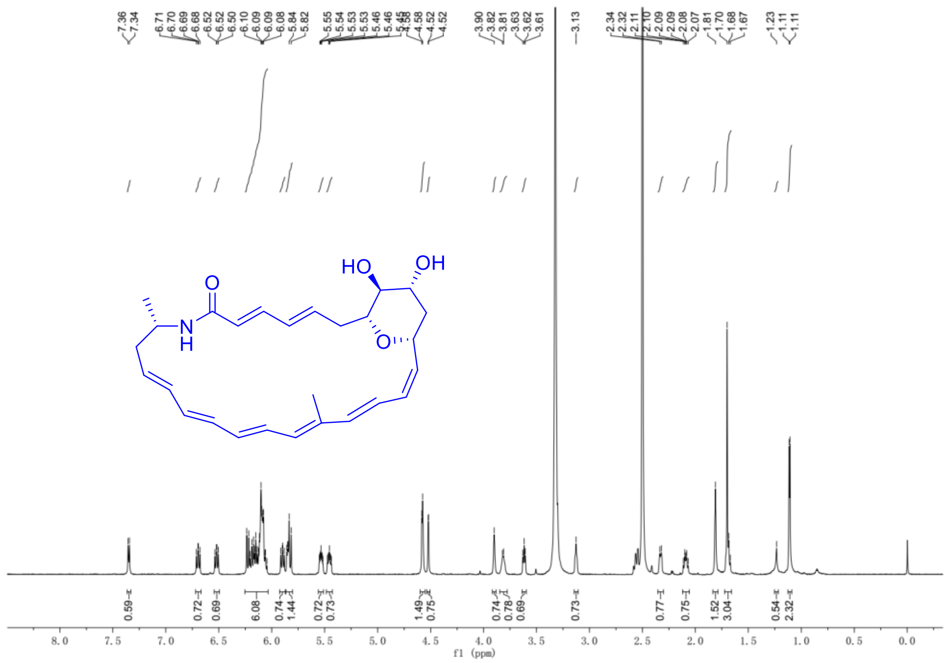

Figure S3 ^{1}H NMR spectrum (800 MHz) of pretilactam (**1**) in DMSO-d_6

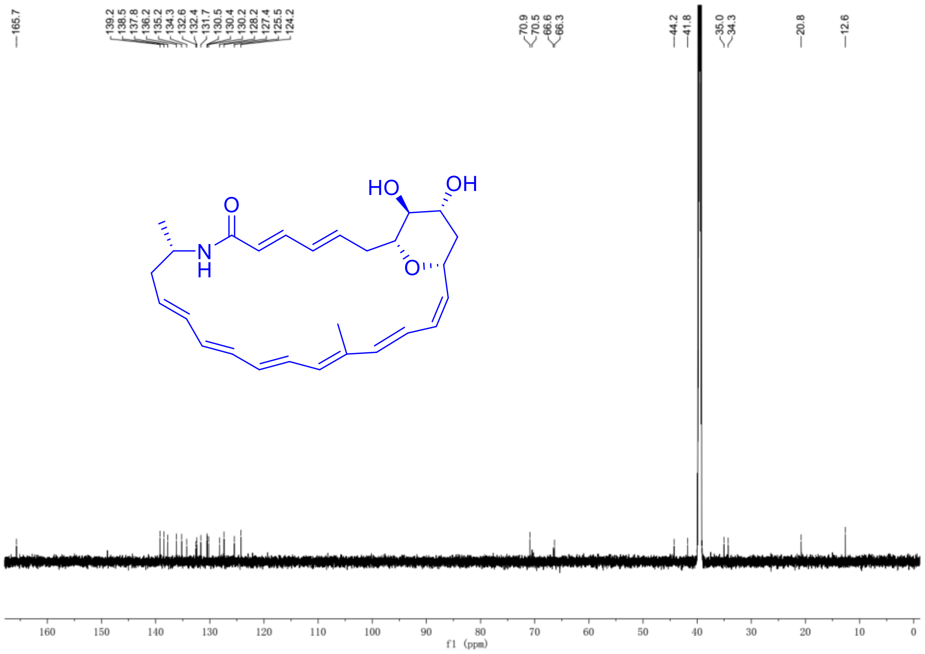

Figure S4 ^{13}C NMR spectrum (200 MHz) of pretilactam (**1**) in DMSO-d_6

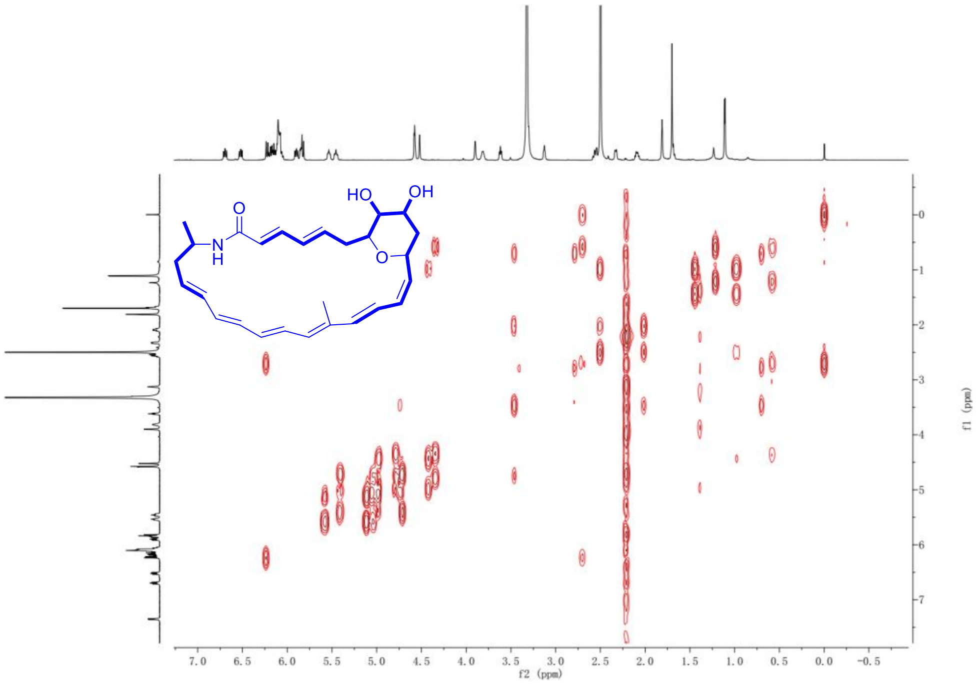

Figure S5 ^{1}H-^{1}H COSY spectrum (800 MHz) of pretilactam (**1**) in DMSO-d_6

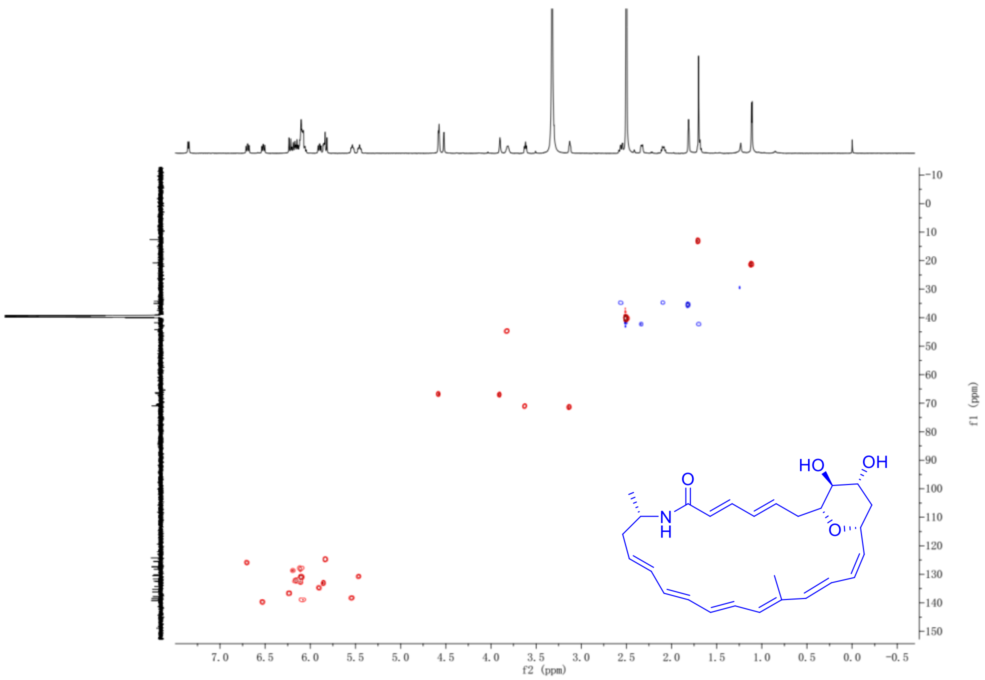

Figure S6 HSQC spectrum (200 MHz) of pretilactam (**1**) in DMSO-d_6

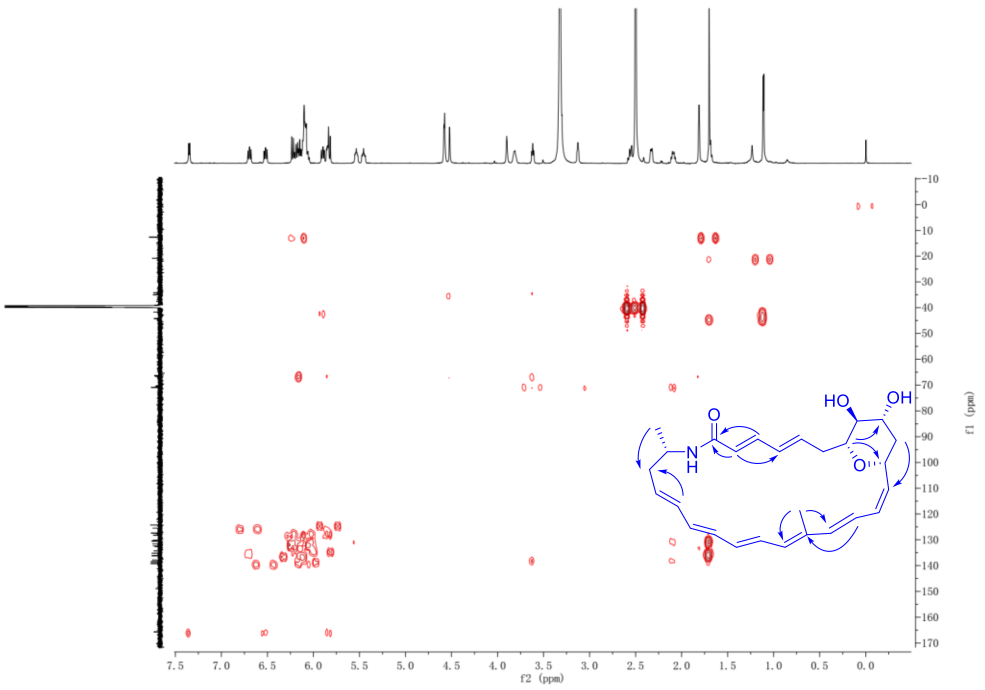

Figure S7 HMBC spectrum (200 MHz) of pretilactam (**1**) in DMSO-d_6

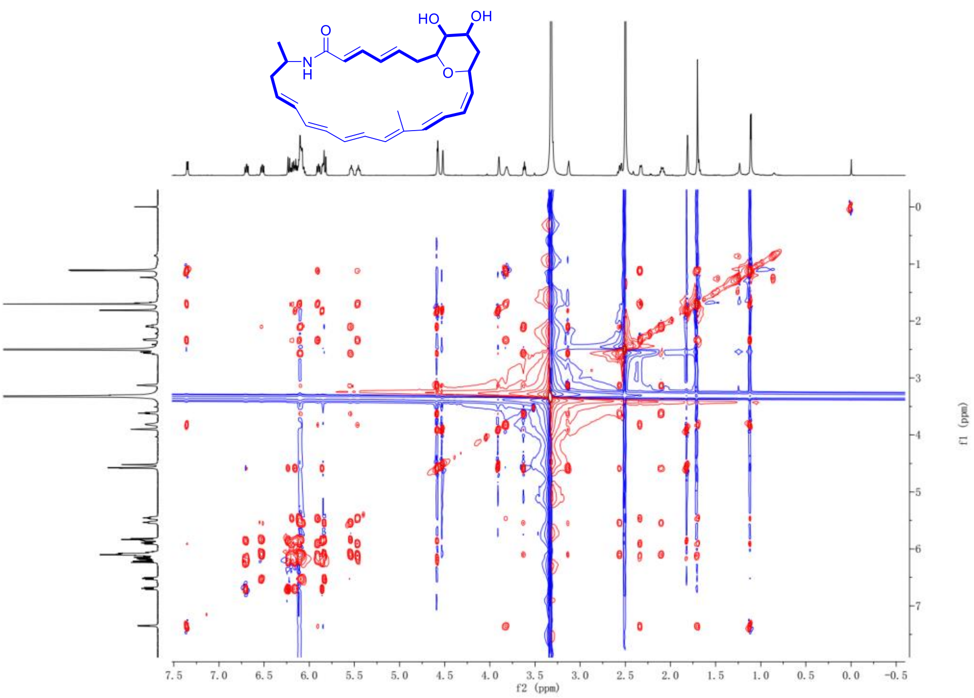

Figure S8 TOCSY spectrum (800 MHz) of pretilactam (**1**) in DMSO-d_6

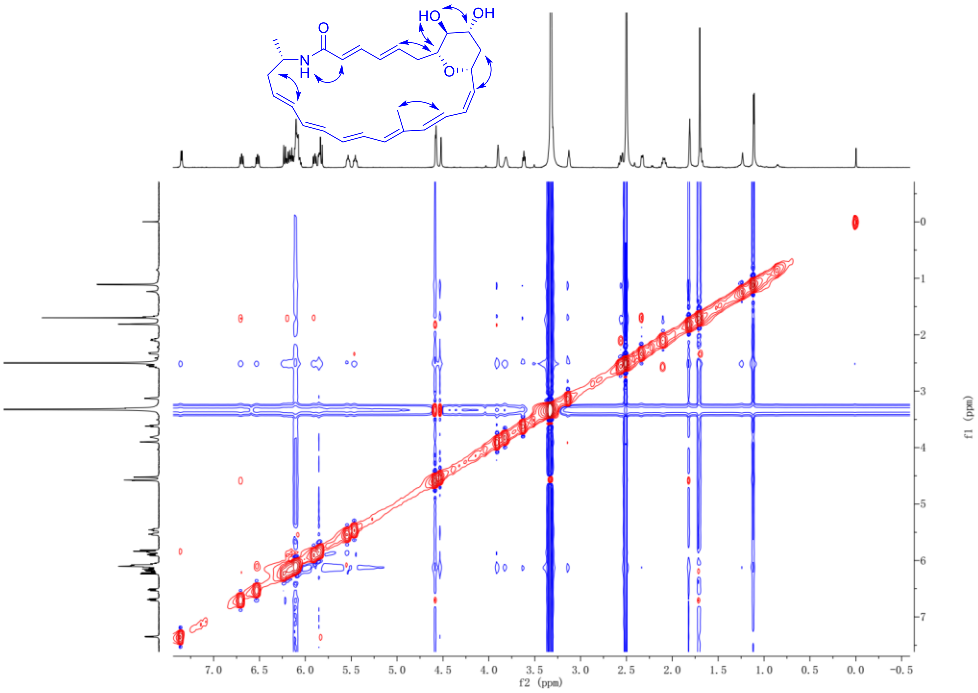

Figure S9 NOESY spectrum (800 MHz) of pretilactam (**1**) in DMSO-d_6

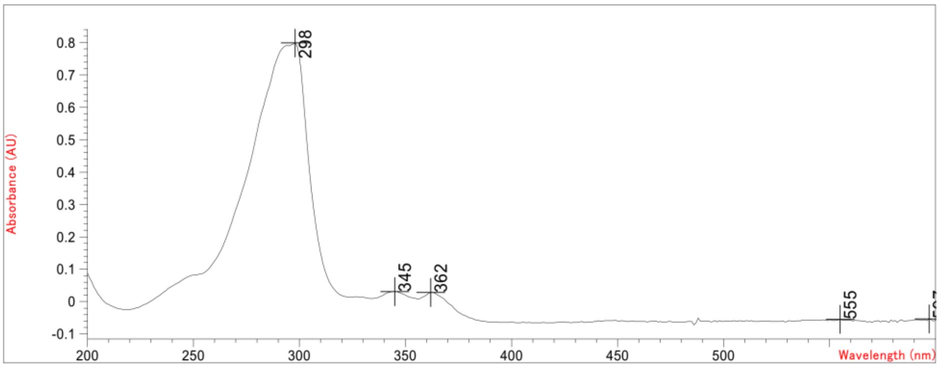

Figure S10 UV spectrum of pretilactam (**1**)

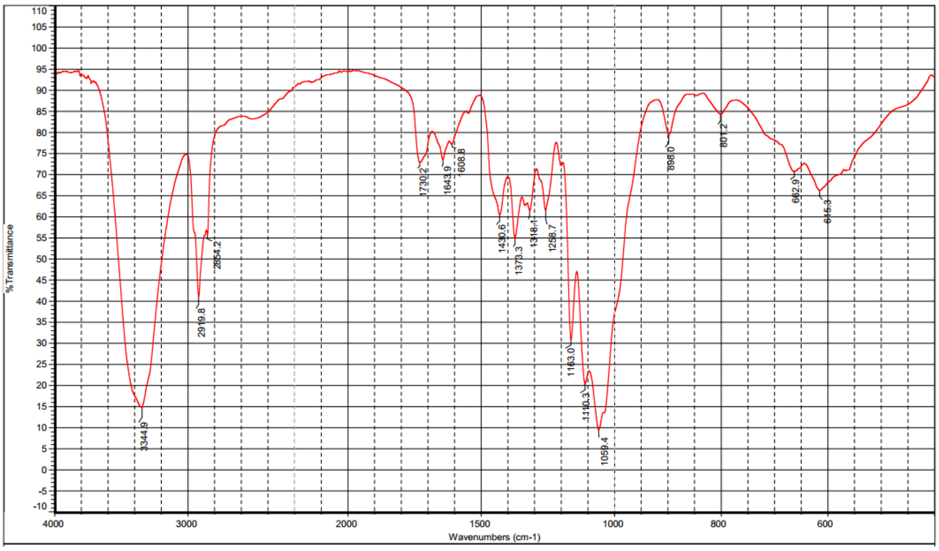

Figure S11 IR spectrum of pretilactam (**1**)
